# Antiasthmatic Effects of Hesperidin, a Potential Th2 Cytokine Antagonist, in a Mouse Model of Allergic Asthma

**DOI:** 10.1155/2011/485402

**Published:** 2011-05-04

**Authors:** Seung-Hyung Kim, Bok-Kyu Kim, Young-Cheol Lee

**Affiliations:** ^1^Institute of Traditional Medicine and Bioscience, Daejeon University, Daejeon 300-716, Republic of Korea; ^2^Department of Herbology, College of Oriental Medicine, Sangji University, Wonju 220-702, Republic of Korea

## Abstract

*Background and Objective*. The features of asthma are airway inflammation, reversible airflow obstruction, and an increased sensitivity to bronchoconstricting agents, termed airway hyperresponsiveness (AHR), excess production of Th2 cytokines, and eosinophil accumulation in the lungs. To investigate the antiasthmatic potential of hesperidin as well as the underlying mechanism involved, we studied the inhibitory effect and anti-inflammatory effect of hesperidin (HPN) on the production of Th2 cytokines, eotaxin, IL-17, -OVA-specific IgE *in vivo* asthma model mice. *Methods*. In this paper, BALB/c mice were systemically sensitized to ovalbumin (OVA) followed intratracheally, intraperitoneally, and by aerosol allergen challenges. We investigated the effect of HPN on airway hyperresponsiveness, pulmonary eosinophilic infiltration, various immune cell phenotypes, Th2 cytokine production and OVA-specific IgE production in a mouse model of asthma. *Results*. In BALB/c mice, we found that HPN-treated groups had suppressed eosinophil infiltration, allergic airway inflammation, and AHR, and these occurred by suppressing the production of IL-5, IL-17, and OVA-specific IgE. *Conclusions*. Our data suggest that the therapeutic mechanism by which HPN effectively treats asthma is based on reductions of Th2 cytokines (IL-5), eotaxin, OVA-specific IgE production, and eosinophil infiltration via inhibition of GATA-3 transcription factor.

## 1. Introduction

The features of asthma are airway inflammation, reversible airflow obstruction, and an increased sensitivity to bronchoconstricting agents, termed airway hyperresponsiveness (AHR), excess production of Th2 cytokines, and eosinophil accumulation in the lungs [1]. The immune cells involved in regulating allergic airway inflammatory responses include monocytes/macrophages, dendritic cells, neutrophils, basophils, mast cells, eosinophils, T and B lymphocytes [2]. Moreover, it is well established that there is a strong interaction between eosinophils and Th2 cells in the asthmatic airways and that Th2 cell-derived cytokines, namely IL-4, IL-5, and IL-13, play critical roles in orchestrating and amplifying allergic inflammation in asthma [3].

CD4+ T cells play a crucial role in immune protection, and they do so through their capacity to help B cells make antibodies, to recruit neutrophils, eosinophils, and basophils to sites of inflammation, and, through their production of cytokines and chemokines, to orchestrate the immune responses [4]. Suppression of cytokine production in activated CD4+ T cells may be useful for the treatment of asthma. 

Th2 cytokines produced by CD4+ T cells including interleukin-4 (IL-4), IL-5, and IL-13 enhance immunoglobulin E (IgE) production, eosinophil accumulation, and IL-13 directly enhances mucus hypersecretion and AHR [5, 6]. Therefore, suppression of Th2 cytokines production in activated CD4+ Th cells may be useful for the treatment of inflammatory immune diseases including asthma.

Hesperidin (HPN, 5,7,3′-trihydroxy-4′-methoxyflavanone7-rhamnoglucoside) belongs to the class of flavonoids called flavanones and is found mainly in citrus fruits and inhibits proliferation of Ramos cells and sensitizes them to doxorubicin-induced apoptosis through inhibition of both constitutive and doxorubicin-mediated NF-*κ*B activation in a PPAR*γ*-independent manner [7]. 

Other biological activities of HPN include inhibition of cell cycle progression in human pancreatic cells [8], reduction of reactive oxygen species and triggering caspase-dependent apoptosis in human polymorphonuclear neutrophils *in vitro* [9], cytotoxic effects on human colon cancer cells, accompanied by DNA fragmentation caspase-3 activation [10], blood pressure [11], reduction of TNF-*α* production [12], anti-inflammatory effects on rodent cell lines and human cell lines [13, 14], an antioxidant effect [15, 16], bone density loss [17], and the reduction of cholesterol [18]. 

However, there have been no reports of the antiasthmatic and anti-inflammatory activities of hesperidin *in vivo *and* in vitro*. The aim of this study was to evaluate the ability of HPN to control Th1- and Th2-type cytokines, various immune cell phenotypes, and other factors. Therefore, using a murine model of asthma, we studied the effect of HPN on airway eosinophil accumulation, Th2 cytokine production, various immune cell phenotypes, and histology.

## 2. Materials and Methods

### 2.1. Animals

Five-week-old female BALB/c mice were obtained from Daehan Biolink Co. LTD. (Eumsung, Republic of Korea). Our study was approved by the committee for animal welfare at the institution Daejeon University. Moreover, all animal procedures were conducted in accordance with the guidelines of the Institutional Animal Care and Use Committee of the Korea Research Institute of Bioscience and Biotechnology (Daejeon, Republic of Korea).

### 2.2. Ovalbumin (OVA) Sensitization and Inhalation

As per the modified protocol previously described [19, 20], OVA (500 *μ*g mL^−1^) in PBS was mixed with equal volumes of 10% (w/v) aluminum potassium sulfate (alum; Sigma) in distilled water, then incubated for 60 min at RT after adjustment to pH 6.5 using 10 N NaOH, and centrifuged at 750 ×g for 5 minutes. The OVA/alum pellet was resuspended to the original volume in distilled water. All mice were immunized on three different days (first day of 2, 3, 4 weeks before inhalational exposure) by intraperitoneal (i.p) injections of 0.2 mL alum-precipitated antigen containing 100 *μ*g of OVA (Sigma-Aldrich Korea, Republic of Korea) bound to 4 mg of aluminum hydroxide (Sigma-Aldrich Korea. Republic of Korea) in PBS. Seven days after the second sensitization intratracheally injected with 250 *μ*g of OVA (on day 21) on the back of the tongue, mice were exposed to aerosolized OVA for 30 min/day, 3 days/week for 5 weeks (at a flow rate of 250 L/min, 1% OVA in normal saline for first 4 weeks and 2% OVA in normal saline for last 1 week). HPN (5 and 1 mg kg^−1^, Sigma-Aldrich Korea, Republic of Korea) and formoterol (1 mg kg^−1^) in solution form were orally administered 3 times a week for the last 5 weeks. One day after the last OVA exposures (2% OVA inhalation), airway hyperresponsiveness was determined and samples (bronchoalveolar lavage fluid, lung cells, and serum) were collected for further molecular analyses.

### 2.3. BALF (Bronchoalveolar Lavage Fluid)

Immediately following assessment of AHR, mice were sacrificed with an i.p injection of sodium pentobarbitone (100 mg kg^−1^). The trachea was cannulated and BAL obtained by washing the airway lumina. Briefly, cells in the lungs were recovered by flushing 1 mL of BAL fluid (1 mM EDTA, 10% FBS, PBS) into the lungs via the trachea. Total cell counts were determined, and 100 *μ*L of fluid was cytospun onto glass slides using a Cytospin centrifuge (Cellspin, Hanil, Republic of Korea; 400 g for 4 minutes). Differential cell counts were performed after staining with a Diff-Quik Stain Set (Baxter Healthcare Corp., Miami, Florida, USA). The supernatant of BALF was stored at −25°C for determination of cytokine levels.

### 2.4. Digestion of Pulmonary Tissue and Cells Preparations

Single cell suspensions from lung tissues and BALF were isolated by mechanical disruption in RPMI 1640 medium supplemented with 2 mM L-glutamine, 100 U/mL penicillin, 100 *μ*g mL^−1^ streptomycin, 50 *μ*M 2-mercaptoethanol, 20 mM HEPES, and 2% heat-inactivated fetal bovine serum (FBS, GIBCO, Grand Island, NY). Briefly, the lungs were removed from thoracic cavity. After mincing using sterile scalpels, the tissue was incubated in PBS containing 1 mg/mL Collagenase IV and 2 mg mL^−1^ Dispase for 40 min at 37°C in a sterile polypropylene tube. After incubation, lung tissue was vigorously pipetted up and down to further dissolve remaining tissue clumps and then filtered using a 70 *μ*m cell-strainer (Falcon, Le Pont de Claix, France). The total number of cells was counted manually using a hemocytometer chamber (Fisher). Between 2 and 4 × 10^3^ cells were spun onto glass slides (Cytospin centrifuge, Cellspin, Hanil, Korea; 400 g for 4 minutes). Differential counts were done according to standard morphologic criteria. 

### 2.5. Determination of Airway Hyperresponsiveness (AHR)

Airway hyperresponsiveness in mice was estimated using a previously described method with modifications [20, 21]. A Buxco system (Biosystem XA; Buxco Electronics Inc, Troy, Conn) was used to evaluate the extent of airway constriction in different groups of mice following the protocol described previously.

Penh is equal to Pause × PEF/PIF, where Pause = (Te − Tr)/Tr (PIF, peak inspiratory flow; PEF: peak expiratory flow; Te, expiratory time; Tr, relaxation time). In this experiment, mice were aerosolized with OVA for 30 min/day, 3 days/week for 5 weeks. At 24 hours after the final inhalation, mice were given aerosolized normal saline, followed by 3.15, 6.25, 12.5, 25, and 50 mg/mL methacholine (Sigma) serially. Airway reactivity was then monitored for 30 minutes. Differences of Penh value between groups were evaluated using an unpaired Student's *t*-test.

### 2.6. Hematoxylin-Eosin (H&E), Masson-Trichrome (M-T), and Periodic Acid-Schiff (PAS) Staining

BALB/c mice were injected, inhaled, and sprayed with OVA for 5 weeks (three times a week) to induce asthma. Two experimental groups were treated with different concentrations of HPN for the later 5 weeks (5 times/week). At the end of the experiment, the lungs were removed and analyzed histologically using a modified protocol previously described [22]. 

Briefly, the lung tissue was embedded in paraffin, then cut into 3 *μ*m thickness sections, stained with H&E solution, M-T solution. The tissue was subsequently mounted and cover-slipped with Dako-mounting medium (Dakocytomation; Denmark Carpinteria CA). The degree of airway inflammatory cell infiltration was scored in a double-blind screen by two independent. The degree of peribronchiole and perivascular inflammation was evaluated by a subjective scale of 0–2, as modified protocol described previously [23]. Periodic acid-Schiff (PAS) stain was performed to identify mucus secretion in lung tissue. Frozen sections (30 mm in thickness) of each tissue were made. Each sample section was mounted on the gelatin-coated slide, stained with PAS reagents, dehydrated, and coverslipped with the permount. The PAS-positive goblet cells were counted manually and normalized to the length of the bronchial epithelial perimeter on the basal side, and expressed as the number of PAS-positive cells per mm of basement membrane.

### 2.7. Antibodies and Flow Cytometric Analysis

All antibodies (such as anti-CD3, CD4, CD8, CCR3, CD69, B220, CD23, CD11b, Gr-1, etc.) for flow cytometric analysis were purchased from Becton Dickinson (BD) PharMingen (San Diego, CA). Cells from lung tissues and BALF were stained with the indicated antibodies in staining buffer (PBS containing 1% FBS and 0.01% NaN_3_) for 10 min on ice, and analyzed by two-color flow cytometry on a FACSCalibur using CellQuest software (BD Biosciences, Mountain View, CA).

### 2.8. Enzyme-Linked Immunosorbent Assay (ELISA)

Interleukin (IFN-*γ*, IL-4, IL-5, IL-13, IL-17, eotaxin, etc.) production in BALF and anti-OVA IgE in serum of the indicated mice (*n* = 5) was measured by ELISA according to the manufacturer's instructions with a monoclonal antibody-based mouse interleukin ELISA kit (R&D system). OVA-specific IL-4 and IFN-*γ* production from spleen cells were suspended in RPMI 1640 medium supplemented with 2 mM L-glutamine, and 5% fetal bovine serum. The spleen cells were then cultured for 48 hrs at a concentration of 1 × 10^5^ cells/well in 96-well culture plates (Corning Inc, Cambridge, Mass) with or without 1 ug mL^−1^ of OVA in a humidified atmosphere of 5% CO_2_ in air at 37°C. The culture supernatants were collected and assayed for IFN-*γ* and IL-4 antibodies induced by OVA using ELISA. All data represent the mean and standard deviation from at least three separate determinants and were compared using a analysis of variance (ANOVA).

### 2.9. Isolation CD4+ T Cells

As previously described [24], splenocytes were isolated from naive BALB/c mice. Cells were enriched for CD4+ cell populations by first staining the cells with anti-CD4 (BD PharMingen, Calif, USA). CD25-cells were isolated from this population by first staining with fluorescein isothiocyanate- (FITC-) conjugated anti CD25 mAb (BD PharMingen) followed by incubation with magnetic-activated cell-sorting anti-FITC beads (Miltenyi Biotec, Auburn, Calif, USA). CD4+ T cells were selected on a (CS) column, and the flow-through was collected as CD4+ T cells. Isolated cells were activated by overnight incubation on 24-well plates coated with 1 *μ*g/mL anti-CD3 (Serotec.), 1 *μ*g/mL anti-CD28 (Serotec.), and with HPN (10, 1 *μ*g/mL) added to RPMI medium supplemented with 1 unit/mL penicillin, 1 *μ*g/mL streptomycin, 20 Mm L-glutamine, 50 mM-mercaptoethanol, and 5% FBS for 48 hours after stimulation.

### 2.10. Intracellular Staining for GATA-3 and IL-17

For GATA-3 (PE mouse anti-GATA3; BD Pharmingen), IL-17 (PE-rat-anti-mouse IL-17; BD Pharmingen) intracellular staining, isolated CD4+ T cells were stimulated in culture medium containing rIL-6 (100 ng/mL; R&D Systems), TGF-*β* (20 ng/mL; R&D Systems), and monensin (GolgiStop, 1 mL/mL, BD Biosciences) in a cell incubator with 10% CO_2_ at 37°C for 4 h. After staining surface markers, cells were fixed and permeabilized using Cytofix/Cytoperm and Perm/Wash buffer (BD Biosciences) according to the manufacturer's instructions.

### 2.11. Statistical Analysis

Data were analyzed by one-way analysis of variance (ANOVA) or unpaired Student's *t*-test followed by Dunnett's multiple comparison test (SPSS version 14.0 statistic software). The difference between the normal group and the control group (OVA + vehicle) was clearly distinguished, and for this reason, statistical significance between the normal group and the control group was not shown in the figures and tables to put an emphasis on the statistical differences between the experimental groups and the control group. Results (presented as mean ± standard error of mean) were considered statistically significant if *P* values were <.05 (*), < .01 (**), or <.001 (***).

## 3. Results

### 3.1. Inhibitory Effect of HPN on Airway Hyperresponsiveness (AHR)

**Figure 1:**
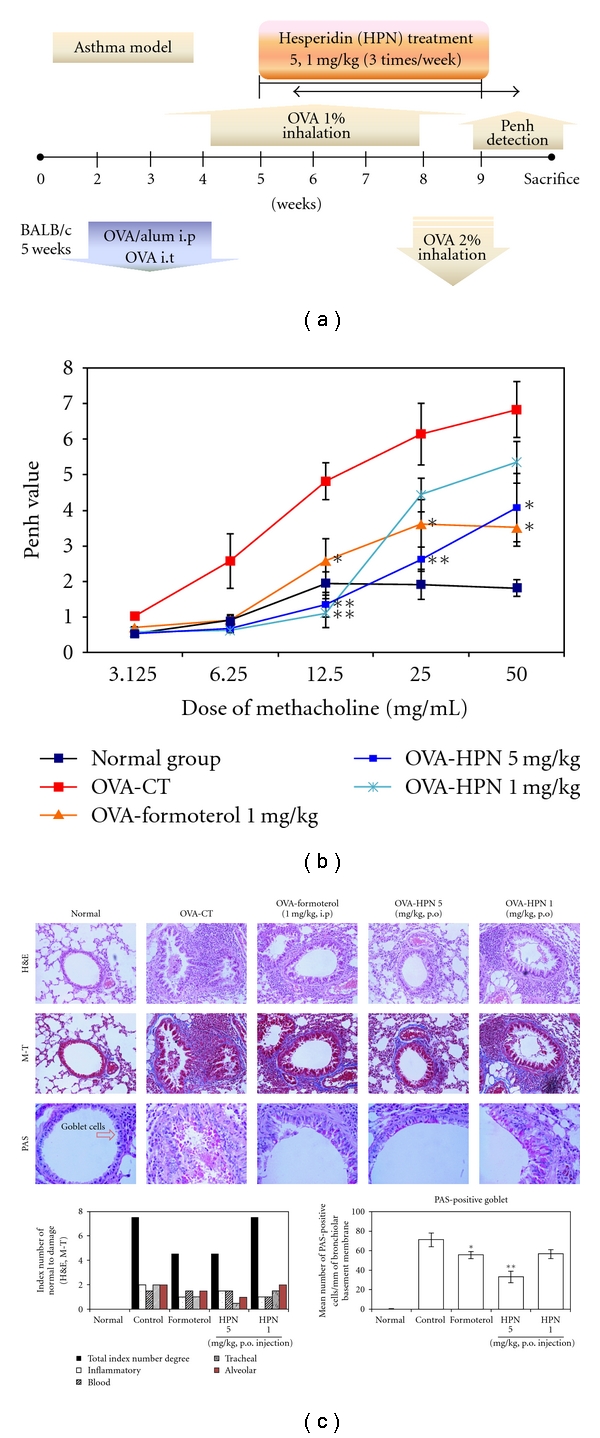
(a) Schematic diagram of methacholine-induced AHR in the sensitization protocol. (b) PenH was measured with a Buxco box, as described in Materials and Methods. *P < .05, **P < .01 for control goup versus HPN-treated groups (c), effect of HPN on histology of lung tissue (H&E, M-T, and PAS staining) in lung cells of the OVA-induced murine model of asthma. H&E: hematoxylin-eosin stain, M-T: Masson trichrome stain, PAS: Periodic acid-Schiff stain, N: normal BALB/c mice, CT (control): Ovalbumin inhalation + vehicle, OVA + formoterol (1 mg/kg), OVA + HPN (5, 1 mg/kg).

 In order to evaluate the inhibitory effect of HPN on airway hyperresponsiveness, total pulmonary airflow in mice was estimated using whole-body plethysmography. PenH was measured using a Buxco system on day 1 after final inhalation, and samples were immediately collected. Methacholine treatment is useful to exhibit the distinct effect of drugs on Penh value by way of inducing AHR. In OVA-sensitized and -challenged mice, the dose-response curve of Penh value was shifted to the left compared with that of normal mice (Figure 1(b)). As shown in Figure 1(b), relative to animals sensitized with OVA (control group), AHR to methacholine was reduced in HPN-treated (5 mg kg^−1^) mice (*P* < .01, *P* < .05) and formoterol treated mice (*P* < .05). However, there was no significant difference between HPN-treated (1 mg kg^−1^) mice and OVA-sensitized and-challenged control mice in their methacholine-induced AHR.

### 3.2. Histological Analysis of Lung Sections

The histopathological investigation of both OVA-challenged mice and HPN formoterol-treated mice showed inflammatory changes when compared with saline-challenged normal mice. Also, we found infiltration of leukocytes in histologic sections of lungs from OVA-challenged control mice, and lung tissue sections from OVA-challenged mice showed a distinct inflammatory infiltrate and erosion in peribronchial and perivascular areas. The peribronchial and perivascular inflammatory infiltrate consisted of eosinophils and mast cells, admixed with lymphocytes. Eosinophil infiltration was mainly observed in the peribronchial regions of the lung. In contrast, histological sections from HPN-treated mice and formoterol-treated mice indicated reduced airway inflammation in lung tissue (Figure 1(c)).

The degrees of goblet cell hyperplasia and mucus hyperproduction were evaluated by means of PAS staining and quantification of PAS-stained cells. The OVA-challenged control mice significantly increased the mean numbers of PAS-positive cells when compared with saline-challenged normal mice. 

In particular, there were greater reduction in the mean number of PAS-stained goblet cells in the HPN-treated (5 mg kg^−1^) and formoterol-treated asthma mice than OVA-sensitized/challenged mice (Figure 1(c)).

### 3.3. Inhibitory Effect of HPN on Airway Eosinophil Accumulation and Influx of Inflammatory Cells into Lung and BALF

The number of total leukocytes in the BALF obtained from the PBS saline challenged group was 0.95 ± 0.05 × 10^7^ cells, indicating that few eosinophils were detected in this group. On the other hand, the total number of leukocytes (2.0 ± 0.1 × 10^7^) and eosinophils in the BALF cytospin of the OVA-challenged was significantly higher than that in the PBS saline-challenged group.

**Figure 2:**
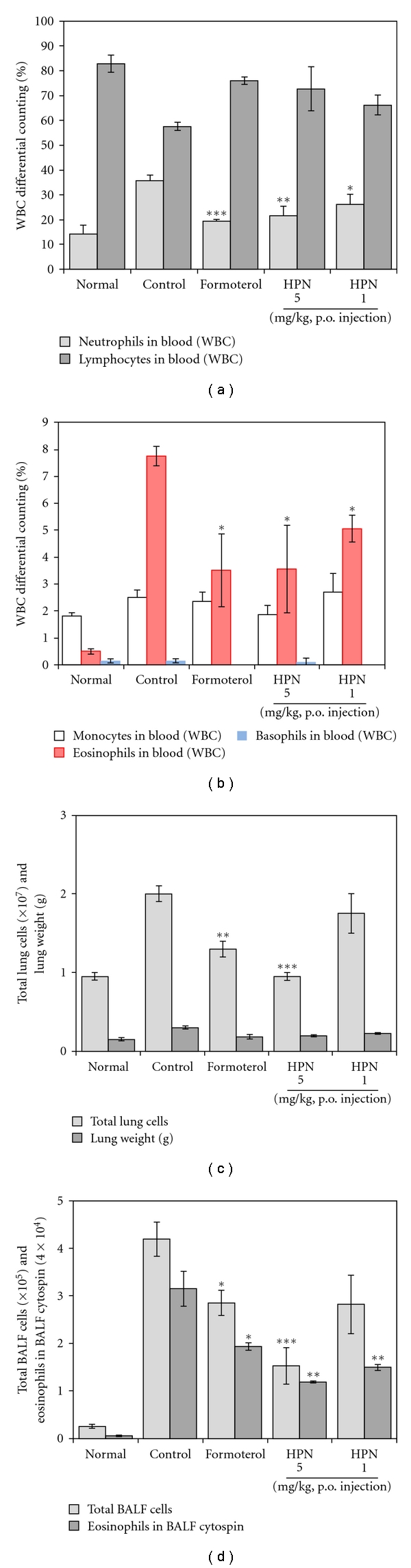
Effects of HPN on total eosinophils, neutrophils, and basophils in blood and total lung cells, total leucocytes, and eosinophils in BALF. As described in Section 2, whole blood was harvested 24 hrs after the last OVA challenge. Total inflammatory cell numbers in blood were counted, and cell classification was performed on a minimum of 200 cells to classify eosinophils and lymphocytes. Results are expressed as mean ± S.E (*N* = 5). Statistical significance between control and treatment groups was assessed by ANOVA (*P < .05, **P < .01, ***P < .001). *N*: normal BALB/c mice, CT: Ovalbumin inhalation+vehicle, formoterol: OVA + formoterol (1 mg/kg), OVA + HPN (5, 1 mg/kg).

The total number of leukocytes were significantly reduced in HPN-treated (5 mg kg^−1^) and formoterol-treated mice compared with control mice, and the number of total lung cells were also significantly reduced in HPN-treated mice (Figure 2(c)). HPN (5 mg kg^−1^) also decreased the absolute number of eosinophils in BALF (Figure 2(d)).

### 3.4. Inhibitory Effect of HPN on Absolute Number of Immune Cell Subtypes in Murine OVA-Induced Asthma Lung and BALF

To evaluate the effect of HPN on T cell subtypes flow cytometric analysis was accomplished. The numbers of CD3, CD4, CD8, CCR3, CD69, B220, CD23, CD11b, Gr-1 positive cells in the lungs of OVA-challenged mice were increased compared to the saline-treated group, and generally, each values from HPN-treated mice were significantly lower than those of OVA-challenged mice (Table 1). Formoterol administration resulted in significant reduction in T cell subtypes similarly.

Effects of HPN on leukocyte subsets in lungs and BALF were marked with change in numbers of CD3+ T cells, CD4+ helper T cells, Gr-1+/CD11b+ granulocytes, CD3−/CCR3+ eosinophils, CD3+/CCR3+ Th2 cells, CD3+/CD69+ early activated T cells, B220+/CD23+ B cells in a mouse model of asthma compared to control group, and the deficits in CD3−/CCR3+ eosinophils were accompanied by concurrent decreases eosinophils in BALF cytospin (Figure 2(d)).

HPN and formoterol groups treated with OVA resulted in significant reductions in CD3+ T cells (**P < .01, ***P < .001), Gr-1+/CD11b+ granulocytes (**P < .01, ***P < .001), B220+/CD23+ B cells (*P < .05, **P < .01, ***P < .001) in lung were decreased significantly and CD3-/CCR3+ (**P < .01), B220+/CD23+ B cells (***P < .001) in BALF were also decreased significantly (Table 1).

### 3.5. Inhibition of Th2 Cytokines (In Vivo and In vitro), Eotaxin, and OVA-Specific IgE Production in BAL Fluid and Serum

The effect of HPN and formoterol on Th2 cytokines and eotaxin protein levels was examined in BALF. 

**Figure 3:**
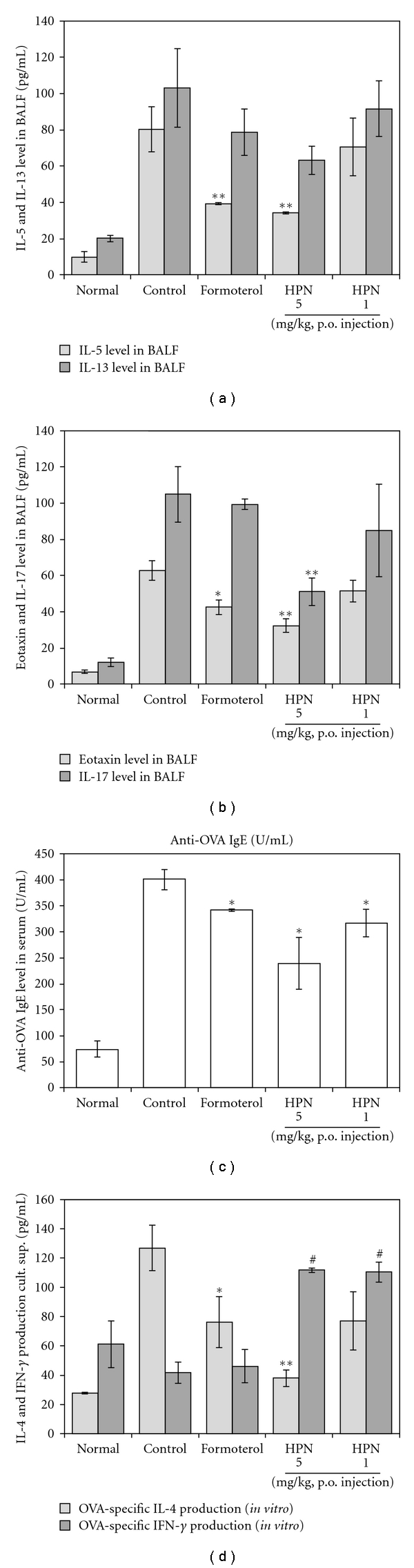
((a), (b), (c)) Effect of HPN on Th2 cytokines (IL-5, IL-13), Th17 cytokine (IL-17), eotaxin in BALF, and OVA-specific IgE in serum. (d) Immunomodulatory effects of HPN on OVA-specific Th1/Th2 cytokines production in spleen cells (described in Section 2). Results are expressed the mean ± S.E (*N* = 5). Statistical significance between control and treatment groups was assessed by ANOVA (*P < .05, **P < .01, significant increase; ^#^ *P* < .05). *N*: normal BALB/c mice, CT: Ovalbumin inhalation+vehicle, formoterol: OVA + formoterol (1 mg/kg), OVA + HPN (5, 1 mg/kg).

As shown in Figures 3(a) and 3(b), IL-5, IL-17, and eotaxin levels were significantly reduced in HPN-treated (5 mg kg^−1^) mice. An important component of allergic asthma model is the production of OVA-specific IgE. Therefore, levels of anti-OVA IgE were measured in serum from the OVA-challenged mice, PBS, formoterol- and HPN-treated groups. In our study, OVA-specific IgE levels in serum from OVA-induced asthmatic mice were significantly increased compared with normal mice (PBS only), and HPN-treated mice had significantly reduced OVA-specific IgE (Figure 3(c)). We also measured IL-4, and IFN-*γ* in the culture supernatants were measured by ELISA and found that HPN (5 mg kg^−1^) significantly inhibited Th2 cytokine (IL-4) production in splenocytes (Figure 3(d)) which was accompanied by a concurrent decrease in Th2 cytokine production in BALF (Figure 3(a)).

### 3.6. Effect of HPN on Expression of GATA3 and IL-17 in CD4+ T cells

**Figure 4:**
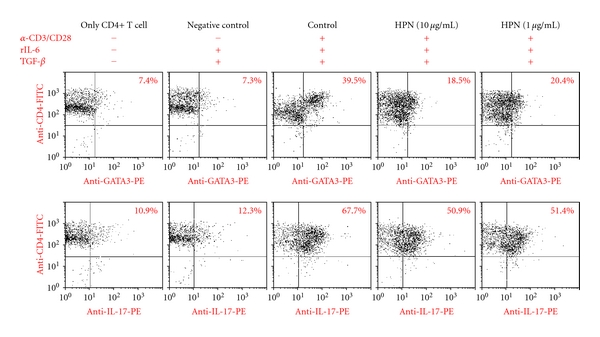
Dot plot pattern of intracellular cytokine staining. *X*-axis: PE intensity of GATA-3 and IL-17. *Y*-axis: FITC intensity of CD4. Isolated CD4+ T cells were cultured without (normal group) or with (stimulated group) anti-CD3, anti-CD28, rIL-6, TGF-beta, and with HPN (10, 1 *μ*g/mL) added to RPMI medium and stained with FITC-conjugated mAb to CD4 and PE-conjugated mAb to GATA-3 or IL-17 (described in Section 2).

As shown in Figure 4, the levels of CD4+/GATA3+, CD4+/IL-17+ double positive cells in CD4+ T cells were significantly increased in control group (39.5%, 67.7%) stimulated with anti-CD3, anti-CD28, IL-6, and TGF-*β* when compared with that of nonstimulated normal CD4+ T cells (7.4%, 10.9%), and negative control (7.3%, 12.3%). Following HPN 10, 1 *μ*g/mL treatment, there was a decrease in the number of CD4+/GATA3+ T cell (18.5%, 20.4%) and CD4+/IL-17+ T cell (50.9%, 51.4%) when compared with that of the stimulated control group.

## 4. Discussion

Asthma is characterized by airway obstruction, which is variable and reversible, and there is chronic inflammation of the respiratory tract, which is mediated by the increased expression of multiple inflammatory proteins, including cytokines, chemokines, adhesion molecules, inflammatory enzymes, and receptors [25]. Th2 cells and their signature cytokines IL-4, IL-5, and IL-13 have key pathogenic roles in asthma [26]. 

Therapeutic agents that may be used in the treatment of asthma are numerous. Anti-IL-5 inhibits eosinophil adhesion, infiltration, and mediator release [27]. Eosinophilia is driven by allergen-activated Th2 cells that generate large amounts of Th2 cytokines (such as IL-4, IL-5, and IL-13). IL-5 is the most critical cytokine mediating increased eosinophil differentiation, activation, and survival [28]. The recruitment and activation of eosinophils appear to be controlled by the release of cytokines such as IL-5 and chemotactic agents such as eotaxin from Ag-stimulated T lymphocytes [29]. It has been suggested that IL-5 and eotaxin may collaborate in the regulation of blood and tissue eosinophilia in mice. 

Although the exact mechanism underlying the biological efficacy of HPN remains elusive, the anti-inflammatory, antioxidant, antidepressant, antimetastatic, and immunomodulatory effect were reported. However, its influence on asthma model has not been studied so far. In the previous study, it was shown that hesperidin at various dosages ranging from 0.3, 5, and 50 mg/kg body weight once daily by i.p injections possessed pharmacological effect [30]. Moreover, HPN attenuates LPS-induced hepatotoxicity possibly by preventing cytotoxic effects of NO and oxygen free radicals [31]. In our preliminary study, HPN treatment (5, 1 mgkg^−1^) did not cause toxic effects on alanine aminotransferase (ALT) and aspartic acid transaminase (AST) levels (data not shown). Therefore, we used the above dose for studying its effect on this model. 

Formoterol, given after the allergen challenge, has a marginal inhibitory effect on the eosinophil influx and inhibits the allergen-induced airway increased airway sensitivity to aerosolized methacholine [32]. At present, formoterol/budesonide is recommended for use as both maintenance and reliever therapy in patients with moderate to severe asthma [33]. We used it as a positive control which plays a critical role for immunosuppressants.

Recruitment of eosinophils to the airways is a characteristic of asthma, and the degree of eosinophilia is correlated with the severity of this disease. These cells are often considered to play a major role in inducing airway hyperresponsiveness (AHR) [34]. Eosinophilic inflammation is regulated to a major extent by activated T lymphocytes in the airways that secrete the Th2 cytokine IL-5 [35]. This cytokine is an important mediator in the regulation of eosinophilic inflammation through effects on the proliferation, differentiation, and activation of eosinophils, as well as providing a signal for the rapid mobilization of eosinophils from the bone marrow [36]. 

HPN prevented the development of AHR (Figure 1(b)), airway eosinophilia (Table 1), lung inflammation (Figure 1(c)), and decreased Th2 cytokine levels (Figure 3(a)) in BAL fluid. These results demonstrate that HPN has profound regulatory effects on the development of lung allergic responses in the OVA-induced asthma model. Moreover, the regulatory effects exhibited by HPN were accompanied by the production of IL-4 *in vitro* assay (Figure 3(d)). 

Asthma produces immune abnormalities in a wide variety of cell populations. Thus, another goal in asthma research includes the evaluation of specific cell subpopulations. Immunophenotyping by flow cytometry showed a similar pattern as total lymphocyte numbers in BALF and lung. 

As previously described in results, effects of HPN on leukocyte subsets in lungs and BALF were marked with change in numbers of CD3+ T cells, CD4+ helper T cells, Gr-1+/CD11b+ granulocytes, CD3−/CCR3+ eosinophils, CD3+/CCR3+ Th2 cells, CD3+/CD69+ early activated T cells, and B220+/CD23+ B cells in a mouse model of asthma compared to control group (Table 1), and the deficits in CD3-/CCR3+ eosinophils were accompanied by concurrent decreases eosinophils in BALF cytospin (Figure 2(d)). HPN also inhibits B cell-dependent production of OVA-specific IgE in serum (Figure 3(c)), which is correlated with the result of B220+/CD23+ B cells in lung and BALF.

It was recently suggested that a transient activation-induced CD69 surface expression may be important for regulating T cell trafficking [37]. Moreover, CD69 might affect the immune response during T-cell differentiation, involving immunoregulatory cytokines that include, but might not be limited to, TGF-*β*, which controls T-cell differentiation [22]. 

CC chemokine receptor 3 (CCR3) is expressed on eosinophils, MC, basophils, and a subset of human Th2-like T lymphocytes [38]. Eosinophils are attracted, via their CCR3, in response to chemoattractants such as eotaxin released in the airways of asthmatics [39]. Inhibition of pulmonary eosinophilia by blocking the CCR3 receptor with antagonists may lead to a reduction in the inflammation and the airway responsiveness in asthma. This approach is investigated by numeral research groups. 

Moreover, eosinophils are one of the cell types known to express Gr-1, therefore eosinophil populations may constitute a substantial portion of the CD11b+ Gr-1+ populations. Our results (Table 1) showed that Gr-1+ cells were increased with OVA challenge but significantly decreased with HPN-treated mice. 

Eotaxin is a potent and specific eosinophil chemoattractant [40] that also activates eosinophils, increasing both leukotriene C4 synthesis and eosinophil peroxidase activity [41]. Eotaxin seems to be most important in the early phases of eosinophil recruitment after allergen challenge [42]. Eotaxin has been previously implicated in allergen-induced AHR [43]. However, the relationship between eotaxin and AHR is complex. In many cases, eotaxin caused substantial airway eosinophilia and in conjunction with IL-5 caused an even more marked increase in eosinophils. 

We observed significant correlations between eotaxin, IL-5 levels, and CCR3 expression on eosinophils. We hypothesized that HPN and formoterol prevent AHR by downregulating eotaxin and IL-5 expression and, in so doing, by reducing eosinophilia. 

A recent study has shown that in a Th2 environment with a dominance of IL-5, a continuous production of eosinophils occurs in a mouse model of allergic inflammation [44]. However, the T cell diversity has been expanded to several subpopulations, including T helper 17 (Th17) cells, suggesting that the mechanism is more complicated [45]. Interestingly, cotransfer of Th17 cells with Th2 cells enhances the expression of eotaxin-1 in the lung, and the neutralization of eotaxin-1 prior to the inhaled antigen challenge decreases the eosinophil recruitment into the airways of the mice transferred with a combination of Th2 cells and Th17 cells [46]. In our result, the mechanism by which antigen-specific Th17 cells achieve suppression of effector T cells is not understood. Therefore, our results do not give us information about the functional status of the above cells, that is, Th2 and Th17 cytokine production can differ because of activation status of some cells. Therefore, our result can partly explain the reported inhibition about the Th2 cytokine (IL-5), eotaxin, and IL-17 protein levels. A possible explanation can be that in each specific mechanisms, the one or the other population can predominate and characterize eventually the final result.

Commitment of CD4+ Th cells into effector Th2 cells is controlled by specific transcription factors GATA-binding protein 3 (GATA-3). GATA-3 is required for the direct activation of Th2 cytokines IL-4, IL-5, and IL-13 for Th2 cell development [47]. 

Various transcription factors such as c-maf, GATA-3, NFAT, and STAT6 have been shown to induce or augment Th2 cytokine production, although only c-maf and GATA-3 are expressed selectively in Th2 cells [48]. In particular, GATA-3 has been shown to promote expression of several Th2 cytokines, including IL-4, IL-5, and IL-13. It is well known that overexpression of GATA-3 predisposes for Th2-mediated diseases such as allergic asthma whereas activation of T-bet appears to be an essential step for Th1-mediated mucosal diseases such as Crohns, disease. For instance, suppression of GATA-3 expression in the lung would presumably suppress IL-4, IL-5, and IL-13 production concurrently.

In particular, GATA-3 is important for the expression of IL-5 in T cells by transactivation of the IL-5 promoter, but GATA-3 only weakly transactivates the IL-4 promoter directly [49]. 

As shown in Figure 4, the levels of CD4+/GATA3+ double positive cells in CD4+ T cells were significantly decreased in HPN treatment group. It can be partly explained that the suppression of GATA-3 expression in the lung would presumably suppress IL-5 production concurrently. However, IL-4 productions *in vivo* (data not shown) and *in vitro* was shown different pattern. It is unclear why OVA-specific IFN-*γ* production from spleen T cells (Figure 3(d)) was enhanced in GATA-3 inhibited condition, but it is possible that an increase in IFN-*γ* alone may not be sufficient for underexpression of GATA-3 and overexpression of T-bet simultaneously. This result appears to be partly due to other signal pathway or more complicated mechanisms involved. 

GATA-3 and T-bet are essential for cytokine gene activation during Th1/Th2 differentiation, but may not be critical factors in Th1 and Th2 cells. The expression of both T-bet and GATA-3 mRNAs decreased in the lungs of mice after repeated antigen challenges. Moreover, the production of Th1/Th2 cytokines in Th1/Th2 cells may not be directly regulated by T-bet or GATA-3, because there is a discrepancy between the expression of T-bet and GATA-3 genes and Th1/Th2 cytokine levels in the lungs of WT mice after repeated antigen challenges [50]. T-bet is regulated by IL-12 via STAT4 and by IL-27 via STAT1. This demonstrates the complex interplay of cytokines and transcription factors in asthma [25]. Therefore, our investigation was focused on Th2 cytokines and GATA-3 transcription factor in this paper. Although the precise effects of T-bet and GATA-3 in the pathogenesis of airway remodeling in asthma are still not fully understood, our finding of underexpression of GATA-3 in the airways asthma model mice suggests that HPN has a significant inhibitory role in asthma.

Therefore, HPN may reduce Th2 cytokine (IL-5) production and gene expression by inhibition of GATA-3 expression. Our result is not sufficient for the explanation of the precise mechanism, and the mechanism is more complicated. However, it is a possible mechanism that HPN shifts immunity from a Th2 to a Th1 bias in a murine model of asthma. It would be interesting to precisely identify the complicated mechanisms of our results concerning Th1/Th2 transcription factors in future studies. Moreover, additional studies are needed to characterize the precise mechanism of therapeutic action of HPN for treatment of asthma.

## 5. Conclusions

Our data demonstrate that HPN has profound inhibitory effects on airway inflammation in a mouse model of asthma, and this effect was caused by suppression of Th2 cytokines (IL-5), B cell-dependent production of OVA-specific and IgE, eosinophil CCR3 expression, through the inhibition of GATA-3 transcription factor. Hence, HPN may act as a potential Th2 cytokine (IL-5) antagonist and may have a therapeutic effect on allergic asthma. 

## Figures and Tables

**Table 1:** Quantification by means of FACS analysis of various immune cell subtypes in lung and BALF. Absolute numbers of various immune cell subtypes in lung were counted (described in Section 2). Results are expressed as mean ± S.E (*N* = 5). Statistical significance between control and treatment groups was assessed by ANOVA (*P < .05, **P < .01, ***P < .001). *N*: Normal BALB/c mice, CT: Ovalbumin inhalation + vehicle, formoterol: OVA + formoterol (1 mg/kg), OVA + HPN (5, 1 mg/kg).

Cell phenotypes		Normal	OVA-induced asthma mice (Absolute no.)			
in lung and BALF		BALB/c	Control	Formoterol	HPN (5 mg/kg)	HPN (1 mg/kg)
CD3+ (×10^5^ cells)		3.43 ± 0.11	9.47 ± 0.52	5.08 ± 0.37***	3.10 ± 0.59***	6.99 ± 0.41**
CD4+ (×10^5^ cells)		2.03 ± 0.07	7.02 ± 0.77	3.02 ± 0.22**	2.51 ± 0.17**	5.69 ± 0.79
CD8+ (×10^5^ cells)		0.54 ± 0.03	1.74 ± 0.04	1.34 ± 0.71	0.64 ± 0.04***	1.53 ± 0.07
Gr-1+/CD11b+ (×10^5^ cells)		1.45 ± 0.01	4.79 ± 0.38	2.07 ± 0.19**	1.25 ± 0.07***	2.01 ± 0.27**
CD3−/CCR3+ (×10^5^ cells)	Lung	0.47 ± 0.09	4.49 ± 1.01	2.58±0.22	1.92 ± 0.13*	3.01 ± 0.25
CD3+/CCR3+ (×10^5^ cells)		0.17 ± 0.04	1.74 ± 0.10	1.19 ± 0.35	0.91 ± 0.15**	1.44 ± 0.04*
CD3+/CD69+ (×10^5^ cells)		0.48 ± 0.01	3.68 ± 0.78	1.27 ± 0.13*	1.42 ± 0.12*	2.40 ± 0.82
CD3−/CD19+ (×10^5^ cells)		1.08 ± 0.03	2.89 ± 0.01	1.66 ± 0.13***	1.23 ± 0.21***	2.37 ± 0.29
B220+/CD13+ (×10^5^ cells)		0.21 ± 0.07	1.76 ± 0.03	0.88 ± 0.15**	0.42 ± 0.03***	0.95 ± 0.26*

CD3+ (×10^3^ cells)		0.44 ± 0.11	23.94 ± 0.82	10.72 ± 0.60**	**6.88** **±** 1.55**	13.40 ± 4.80
CD4+ (×10^3^ cells)		0.43 ± 0.03	21.23 ± 1.57	9.57 ± 0.78**	7.92 ± 1.65**	15.69 ± 6.08
CD8+ (×10^3^ cells)		0.06 ± 0.03	2.92 ± 0.60	2.35 ± 0.61	0.85 ± 0.40	1.96 ± 0.53
CD3−/CCR3+ (×10^3^ cells)	BALF	0.03 ± 0.02	5.75 ± 1.14	1.62 ± 0.01**	0.53 ± 0.11**	1.14 ± 0.16**
CD3+/CCR3+ (×10^3^ cells)		0.04 ± 0.01	10.79 ± 1.15	5.18 ± 0.44**	2.11 ± 0.87**	6.34 ± 1.45
B220+/CD23+ (×10^3^ cells)		0.12 ± 0.01	6.64 ± 0.15	2.65 ± 0.51***	0.94 ± 0.32***	1.43 ± 0.41***
